# Association of Plasma Metabolites and Salt Sensitivity of Blood Pressure in Chinese Population: The EpiSS Study

**DOI:** 10.3390/nu15030690

**Published:** 2023-01-30

**Authors:** Fengxu Zhang, Yunyi Xie, Xiaojun Yang, Wenjuan Peng, Han Qi, Bingxiao Li, Fuyuan Wen, Pandi Li, Yuan Sun, Ling Zhang

**Affiliations:** Department of Epidemiology and Health Statistics, School of Public Health, Capital Medical University, and Beijing Municipal Key Laboratory of Clinical Epidemiology, Beijing 100069, China

**Keywords:** metabolomics, salt sensitivity, blood pressure, biomarkers, diagnostic efficiency

## Abstract

Background: To identify novel metabolites associated with salt sensitivity of blood pressure (SSBP) in Chinese Han population. Methods: A case-control study was conducted with 25 salt sensitive (SS) and 26 salt resistant (SR) participants, which was selected from the Systems Epidemiology Study on Salt Sensitivity of Blood Pressure (EpiSS) study. The modified Sullivan’s acute oral saline load and diuresis shrinkage test (MSAOSL-DST) was conducted to identify SS. Untargeted, ultra-high performance liquid chromatograph-high resolution mass spectrometer (UPLC-HRMS) was conducted and orthogonal partial least squares-discriminate analysis (OPLS-DA) and multivariable logistic regression model were used to screen the metabolites related to SS, mixed linear regressions models were used to examined the association of SSBP with metabolites during saline load period and diuresis shrinkage period. Receiver operating characteristic (ROC) curve analysis was performed. The area under the curve’s (AUC) sensitivity and specificity were calculated to identified metabolites biomarkers for SS. Results: There were 39 differentially expressed metabolites (DE-metabolites) between SS and SR. Thirty-five and four of DE-metabolites were inversely or positively associated with SS, respectively. Four biochemical pathways demonstrated significant enrichment for identified metabolites. In single-metabolite analyses, L-Glutamine displayed the best diagnostic performance (AUC = 0.88, 95% CI: 0.78–0.97). In multi-metabolites analyses, L-Glutamine + Cholesterol ester 22:5n6 combination showed the best diagnostic performance (AUC = 0.96, 95% CI: 0.91–1.00). Adjusted for traditional risk factors, L-Glutamine and Cholesterol ester 22:5n6 explained an additional 38.3% of SS susceptibility. Conclusions: This study provide potential evidence for clarifying the mechanism of SS and provide novel biological insights into salt sensitive hypertension.

## 1. Introduction

Hypertension is a complex disease caused by genetic and environmental factors that affects 1.1 billion people worldwide [1]. Results of the China Hypertension Survey (CHS) show that the number of patients with hypertension in Chinese people over 18 years old is 245 million [2]. Salt is one of the most important environmental factors. Epidemiological studies, animal experiments and clinical trials have displayed a causal relationship between high dietary salt intake and elevated blood pressure [3,4]. The response of blood pressure to dietary salt intake is heterogeneous between individuals, which is called salt sensitive (SS) [5]. SS is a recognized hypertension endophenotype [6,7]. Additionally, the hypertension related to SS is called salt sensitivity hypertension (SSH). SS has become the main feature of hypertension in China [8,9]. Hence, Clarifying the pathogenesis mechanism of SSH and screening reliable biomarkers for rapid identification of SSH are the basis for early prevention and precise treatment, which need to attract extensive attention from the society. Several studies have reported that SS is an important risk factor for hypertension, renal insufficiency and insulin resistance [5,6,7,10,11], which suggested that a reduction in the huge burden of these diseases may possibly be achieved through early detection of SS.

The mechanism of SS promoting essential hypertension is complex, which involves the dysfunction of multiple systems of the body. Additionally, the evidence based on population is lacking, which makes it difficult to evaluate the risk of hypertension caused by SS. Metabolomics, as one of the important “omics”, has provided a comprehensive snapshot of metabolic state, and has been widely used to study the etiology and risk factors of multiple polygenic complex diseases [12,13], including hypertension [14,15]. Metabolomics can amplify the small changes in genes. What is more, it reflects the changes in the internal and external environment of the body [16]. At the same time, metabolite is easier to detect, which is suitable for clinical application. High-throughput nuclear magnetic resonance spectroscopy and mass spectroscopy can be used to metabolite profiling of participants [17]. Considering SSH was a metabolic disorder and had abnormal metabolites, using metabolomics advance our understanding of SS pathogenesis and improving risk prediction by combining new biomarker information other than traditional risk factors [18]. However, there are only two population-based metabonomic studies focus on SS [19,20]. The two studies mentioned above are based on human urine to carry out untargeted metabolomics. Currently, there is a lack of population-based studies on SS based on plasma metabolomics in Chinese people. The detection of SS in the population is of great significance for preventing and diagnosing SSH and reducing the incidence rate and mortality of cardiovascular events [21,22]. However, the current method to determine SS has cumbersome implementation steps, limited accuracy and poor acceptability and is not suitable for the detection of large-scale populations. It is urgent to find a method that can quickly and accurately distinguish salt sensitive (SS) from the population. Moreover, current metabolomics research on SS mostly focuses on animals. 

Bases on the EpiSS cohort we have established, in this study, we aimed to identify plasma metabolites associated with SS in Chinese Han populations and obtain the specific metabolites that highly associated with SS. Then, we aimed to determine whether metabolites could be used in SS risk prediction in addition to traditional risk factors.

## 2. Materials and Methods

### 2.1. Participants 

Participants were selected from the System Epidemiology Study on Salt Sensitivity of Blood Pressure (EpiSS) cohort study, which was registered in the Chinese Clinical Trial Registry (No:ChiCTR-EOC-16009980, http://www.chictr.org.cn/showproj.aspx?proj=15690, accessed on 23 November 2016) and details can be found in the previously published protocol [23]. In short, 25 SS and 26 age (±5 years), gender matched SR participants were involved in the untargeted metabolomics study. All participants were 45–70 years old. Participants with cardiovascular disease, kidney disease and malignant tumors were excluded. Capital Medical University’s ethics committee approved this study and all participants provided written informed consent.

### 2.2. Determination of SS

According to the previous study, the Sullivan’s acute oral saline load and diuresis shrinkage test (MSAOSL-DST) modified by Li Yuming was used to determine the SS [24,25], the details was described in the protocol of EpiSS as the following steps [26]: First, the baseline blood pressure (BP_0_) was measured at the begin of this study. Second, the participants were asked to drink 1000 mL of saline solution on an empty stomach in the morning and drink it within 30 min. The second time BP (BP_1_) was measured after two hours from the time they finished drinking saline, which was called saline load period. Then, all participants were asked to take 40 mg furosemide immediately. The third time, BP (BP_2_) was measured two hours after oral administration of furosemide. Mean arterial pressure (MAP) was calculated at three time points including baseline, two hours after high salt intervention and two hours after diuresis. _Δ_MAP_1_ was defined as MAP_1_ − MAP_0_ and _Δ_MAP_2_ was defined as MAP_2_ − MAP_1_. Individuals with _Δ_MAP_1_ ≥ 5 mmHg or _Δ_MAP_2_ ≤ −10 mmHg were defined as SS and the other participants were salt resistant (SR) [25].

### 2.3. Data and Sample Collection

Questionnaire survey: the standardized and unified trained investigators conducted a questionnaire survey through on-site face-to-face inquiry, including general information (name, gender, age and education); lifestyle (smoking, alcohol drinking, sleeping and diet survey); and illness (disease history, family history and treatment history).

Physical examination: During the measurement of height and weight, the subjects should be barefoot, bareheaded and dressed in simple clothes. The subjects should be in an upright position with their feet 30–40 cm apart. Body mass index (BMI) = weight/height ^2^ (kg/m^2^).

BP phenotypes measurement: The BP of the right arm in the sitting position was measured (Omron HEM-7118, Osaka, Japan). During the determination of SS, the BP was measured twice at each time point and the average of the blood pressure measured twice was taken as the blood pressure at that time point. Hypertension was defined according to the 2018 Chinese guidelines for the management of hypertension [27] as a SBP ≥ 140 mmHg, DBP ≥ 90 mmHg, or use of antihypertension medication.

Blood collection: During the EpiSS baseline, trained nurses collected the subjects’ fasting 8-h venous blood and 5 mL blood samples were collected using EDTA blood collection tubes and coagulating blood collection tubes manufactured by BD Company. The blood samples are stored in the −80 °C refrigerator. Repeated freezing and thawing are avoided during the process of sample storage and transfer. The blood samples were centrifuged and sent to the company for unified detection of fasting blood glucose (FBG), total cholesterol (TC), triglycerides (TG), low-density lipoprotein cholesterol (LDL-C) and high-density lipoprotein cholesterol (HDL-C).

Urine collection: 1 mL urine sample was taken at the time point when the patient got up in the morning with an empty stomach, and the concentration of urine sodium and potassium was detected. Within one week of the salt sensitivity determination test, the subjects were required to collect urine for 24 h under the daily diet status.

According to the 24 h urinary sodium excretion, the formula for evaluating the daily salt intake is as follows [28]:

$$Salt\;intake\;\left(g/\mathrm{day}\right)=\frac{24hUNaE\times 24hUV\times M\left(NaCl\right)}{1000}$$

24 *hUNaE* = 24 h urinary Na^+^ excretion value (mmol/L)

24 *hUV* = 24 h urine volume (L)

*M* = molecular mass

### 2.4. Metabolomics Profiling

Untargeted metabolomics analysis was conducted on Ultimate 3000 ultra-high performance liquid chromatograph coupled with Q Exactive^TM^ quadrupole-Orbitrap high resolution mass spectrometer (UPLC-HRMS) system (Thermo Scientific, Waltham, MA, USA) using EpiSS plasma samples that had been stored at −80 °C. In this study, the area under the peaks curve was used to quantify metabolites. To correct variation resulting from instrument tuning differences between days, a normalization step was performed. A proportional correction was applied to each compound by rescaling its median to one and normalizing each data point proportionally. The results of metabolomic analysis detected and quantified 970 metabolites, including 944 known biochemical compounds and 26 unnamed compounds, which were marked with “X” followed by numbers (e.g., X-12345).

### 2.5. Quality Control

We took the metabolite extract of each sample in equal amount, mix and centrifuge and prepare pooled Quality control (QC) samples for testing [29]. Four QC samples were evenly inserted before, during and after each analysis sequence for analysis quality control. The spearman correlation coefficient of four QC samples were calculated.

### 2.6. Statistical Methods

Statistical analyses were performed using SPSS 25.0 (SPSS, Inc., Chicago, IL, USA) and R software (version 4.1.3). *p*-value < 0.05 was considered statistically significant. Measurement data are presented as the mean ± standard deviation. *t*-test were used to analyze normally distributed data. Nonnormally distributed data were analyzed using a Wilcoxon rank sum test. The metabolome data were Log2 transformed for final statistical analysis. The Orthogonal Partial Least Squares-Discriminant Analysis (OPLS-DA) was conducted with SIMCA-P software [30] (Umetrics, Sweden). *t*-test, *p* value FDR adjust, and metabolic pathway analysis was conduct on MetaboAnalyst (https://www.metaboanalyst.ca, accessed on 12 December 2022). The association between each metabolite and SS was analyzed using logistic regression analysis adjusted for age, gender, smoking, drinking, LDL-C, HDL-C and hypertension. Odds ratios (ORs) and 95% confidence intervals (CI) represent the risk of SS. Mixed linear regression models were used to examine the association of SSBP with metabolites during saline load period and diuresis shrinkage period, ΔMAP_1,2_ as the dependent variable, adjusting for baseline age, gender, smoking, drinking, LDL-C, HDL-C and hypertension again. To confirm the robustness of the results, we included other potential confounders (e.g., BMI and salt intake) as sensitivity analysis. Receiver operating characteristic (ROC) curve analysis was performed. The area under the curve (AUC), sensitivity and specificity were calculated to identified metabolites biomarker for SS.

To compare the diagnostic accuracy of different combinations of metabolites, a Monte Carlo cross validation model were constructed, using Linear Support Vector Machine (SVM) classification method and subsequently calculated the AUC of the ROC curves [31]. Through cross-validation (CV) of repeated random sub-samplings, the algorithm aims to identify important features. To build classification models, the top 5, 10, 15, 25, 50 and 100 important features are used, which are then validated on the samples left out. The significant features are ranked by their frequencies of being selected in the models.

## 3. Results

### 3.1. Characteristics of Participants

We enrolled 51 participants with an average age (mean ± standard deviation) of 57.72 ± 5.42 years and 29.4% males for the untargeted metabolomics analysis. The characteristics of two groups with 25 SS and 26 SR are summarized in Table 1. Variables in the untargeted metabolomics study were comparable between SS and SR except for LDL-C (*p* = 0.012), SBP (*p* = 0.001) and DBP (*p* = 0.001).

### 3.2. Plasma Metabolite Levels in SS and SR

**Figure 1:**
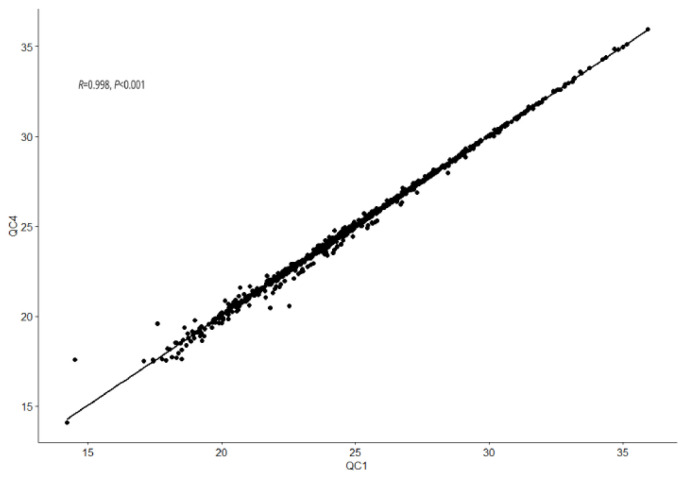
Spearman correlation analysis of the first and last QC samples in the analysis batch. High correlation indicated high data quality of acquired untargeted metabolomics data.

The QC result are shown in Figure 1. In four QC samples, QC1 and QC4 showed the smallest correlation coefficient. However, even for this pair of samples with the lowest correlation, we can still see the high correlation between them in the scatter chart of Spearman correlation analysis (*R* = 0.998, *p* < 0.001), which means that the data quality is excellent.

#### 3.2.1. Metabolites Nominally Associated with SS

**Figure 2:**
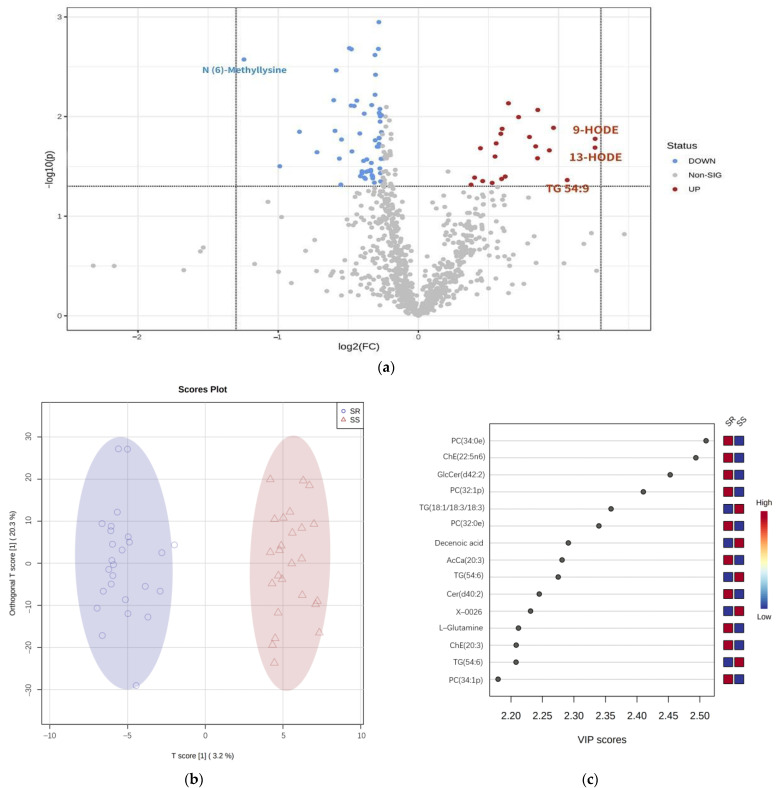
Metabolite signatures differentiate SS and from SR. (**a**): Volcano plot of differentially expressed metabolites. (**b**): OPLS−DA clustering the SS and SR. (**c**): PC 34:0e and ChE 22:5n6 are the most relevant parameters distinguishing SS from SR based on VIP score.

Volcano plot identified 79 differentially expressed metabolites (DE−metabolite) between SS and SR. The metabolites with the greatest changes were Triacylglycerol (TG 54:9) and oxylipins 9/13−hydroxyoctadecadienoic acid (9−HODE/13−HODE), which were increased, and the N (6) −Methyllysine which were decreased (Figure 2a). Furthermore, SS were completely segregated from SR by OPLS−DA (Figure 2b). Glycerophosphocholine (PC 34:0e) and Cholesterol esters (ChE 22:5n6) were identified as the most effective metabolomics in distinguishing the groups of SS from SR according to Variable Importance in Projection (VIP) score (Figure 2c). We found that there are 13 categories (e.g., amino acid and cholesterol esters) of 39 DE−metabolites between the SS and SR groups (*p* < 0.05, Supplementary Table S1). Thirty-five and four of them were inversely or positively associated with SS. While all the metabolites failed FDR corrected significance (*p* < 0.05).

#### 3.2.2. Pathway Analysis

Supplementary Table S2 showed the KEGG biochemical pathways identified among SS metabolites. Sphingolipid metabolism (*p* = 0.003, pathway impact = 0.31), pyruvate metabolism (*p* = 0.004, pathway impact = 0.32), arginine biosynthesis (*p* = 0.017, pathway impact = 0.06) and citric acid cycle (*p* = 0.034, pathway impact = 0.09) demonstrated significant enrichment for identified metabolites (Supplementary Figure S1).

### 3.3. Multiple Logistic Regression Analysis

The results of multiple logistic regression analysis between each 39 metabolites and SS are shown in Table 2. After adjusting for age, gender, smoking, drinking, LDL−C, HDL−C and hypertension, there were still 17 metabolites significantly associated with SS. After adjusting BMI and salt intake, respectively, the results were robust (Supplementary Table S3).

Furthermore, we analyzed the association between these metabolites and SSBP during saline load period and diuresis shrinkage period. Adjusted for age, gender, smoking, drinking, LDL-C, HDL-C and hypertension, there are 14 metabolites remained significant between saline load periods. Nine metabolites remained significant between diuresis shrinkage period (Table 3).

### 3.4. Metabolites Diagnostic Performance by ROC Analysis

#### 3.4.1. Single-Metabolite Diagnostic Performance by ROC Analysis

The diagnostic values of single-metabolite models for SS were evaluated using ROC analyses (Supplementary Table S4). L-Glutamine showed the best diagnostic efficiencies for SS. The AUC, sensitivity and specificity values of L-Glutamine were 0.88 (95% CI: 0.78–0.97), 88% (95% CI: 75–100%) and 77% (95% CI: 61–93%), respectively.

#### 3.4.2. Added Value of Metabolites in the Prediction of SS

**Figure 3:**
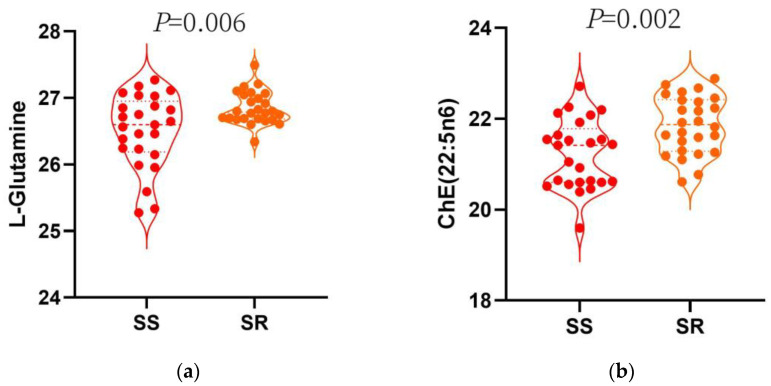

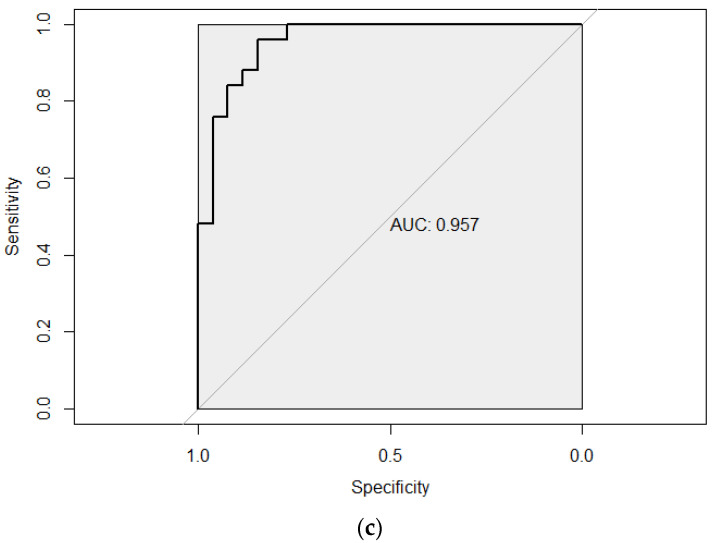
(**a**) Plasma relative level of the L−Glutamine in SS and SR. *t*−test *p* value < 0.05 between the two group. (**b**) Plasma relative level of the ChE 22:5n6 in SS and SR. *t*−test *p* value < 0.05 between the two group (**c**) Receiver Operating Characteristics plot of the model.

Adjusted for age, gender, smoking, drinking, LDL-C, HDL-C and hypertension, a combination of L-Glutamine + ChE 22:5n6 showed the best diagnostic performance in two metabolites model. Plasma relative level of the L-Glutamine and ChE 22:5n6 between SS and SR were shown in Figure 3. The diagnostic efficiency of two-metabolites models was increased when compared with single-metabolite models (Supplementary Table S3), with AUC, sensitivity and specificity values of 0.96 (95% CI: 0.91–1.00), 96% (95% CI: 88–100%) and 85% (95% CI: 71–98%), respectively (Figure 3c). Adding the two metabolites into the model yielded an additional 38.3% of the variance in SS risk, and improved the C-statistic from 0.75 to 0.96 (*p* < 0.0001; Table 4).

#### 3.4.3. ROC Analysis of Metabolites Based on Monte Carlo Models

**Figure 4:**
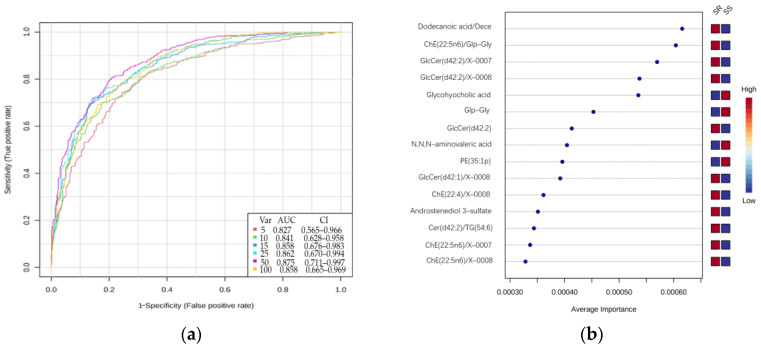
Identification of biomarkers for SS. (**a**): ROC of Monte Carlo models corresponding to the combination of 5 to 100 variables. (**b**): Relative average importance of the different variables chosen by the model.

We used Monte Carlo models to identify biomarkers of SS using metabolites. The top 5, 10, 15, 25, 50 and 100 important features are used to build classification models and calculate the AUC using the combined ROC curves. The numbers of variables were progressively increased to 100 in six different models. The AUC-ROC curves in all cases were greater than 0.82 (Figure 4).

## 4. Discussion

Using an untargeted MS-based metabolomics platform, we found that there are 13 categories (e.g., amino acid) of 39 DE-metabolites between the SS and SR groups. Thirty-five of them were inversely associated with SS and four of them were positively associated with SS. After adjusted for age, gender, smoking, drinking, LDL-C, HDL-C and hypertension, 14 and 9 metabolites remained significant during saline load period and diuresis shrinkage period. Four biochemical pathways, Sphingolipid metabolism, pyruvate metabolism, arginine biosynthesis and citric acid cycle, demonstrated significant enrichment for identified metabolites. Most of these DE-metabolites are not routinely tested in clinical, which provides new insights into the molecularly characterize of SS. Furthermore, L-Glutamine was identified as metabolomics biomarkers for SS. The optimization diagnostic model of SS constructed by L-Glutamine + ChE 22:5n6 had the AUC curve with 0.96 in this study. Considering the cost of testing, a metabolites diagnostic model including L-Glutamine and ChE 22:5n6 is recommended.

SS plays an important role in the occurrence of cardiovascular diseases, researchers have conducted increased research on biomarkers of SS. Including genomics [10], transcriptomics [32] and metabolomics [20,33]. These studies provide potential evidence for clarifying the mechanism of SS. In our study, we found most of metabolites are negative related to SS, which means these metabolites are protective factor of SS, such as L-Glutamine belonging to amino acid. L-Glutamine performs a fundamental role in cardiovascular physiology and pathology [34]. The expression level of L-Glutamine in hypertensive population decreased [35]. SS is an important risk factor for SSH [6,7], the negative association between L-Glutamine and SS may seem reasonable. L-Arginine generates NO through NO synthase to promote cardiovascular health [36,37]. As precursor of L-arginine, L-Glutamine can optimize NO synthesis [34,38]. The increase in NO content can promote the vasodilation function and relieve SSH. These metabolites positive associated with SS, such as Triacylglycerol 54:6, Sphingosine 1-phosphate, Alpha-dimorphcolic acid and 13S-hydroxyoctadecadienoic acid are consistent with previous research results. In patients with early autosomal dominant polycystic kidney disease (ADPKD), the level of 13-hydroxyoctadecadienoic acid (HODE) is increased [39]. ADPKD patients are often accompanied by SSH. The 13-HODE is a marker of oxidative stress and mediators of the inflammatory response [40]. Oxidative stress and inflammatory reaction are closely related to SSH [41,42]. Animal experiments show that continuous high salt diet intervention will cause increase in blood pressure in SS rats, further accumulation of peroxides in renal cortex and medulla and make the kidneys under oxidative stress [43]. Thus, cause the occurrence of SSH [44].

Currently, there are many reports on the identification methods of SS in the population and there is a lack of unified and standardized measurement methods and judgment standards. It is generally divided into acute intravenous salt load test [45] and chronic salt load test [46,47], but they are laborious or costly. Therefore, it is of practical significance to study and screen SS related biomarkers to assist the diagnosis of SS, but there are few relevant reports at present. Our previous studies showed that genetic variants moderately predict SS and hypertensive status [48,49]. Here we tested if metabolites could improve its predictive power. In this study, we found a diagnostic model for SS based on SS related metabolites. In single-metabolites model, L-Glutamine showed the best diagnostic efficiencies for SS (AUC = 0.88, 95% CI: 0.78–0.97). A multi-metabolites model based on L-Glutamine and ChE 22:5n6 showed a significant increase on the diagnostic ability of SS (AUC = 0.96, 95% CI: 0.91–1.00). Finally, we compared the accuracy of using machine method to predict SS and logistic regression method. In this study, the AUC of logistic method is higher than Monte Carlo models. Monte Carlo models create ROC curves based on cross-validation performance of SVM. By contrast, the classical univariate ROC curves are based on the performance measured by testing all possible cutoffs within all data points. Therefore, the ROC calculated from the cross-validated ROC curve is more realistic for prediction purposes, whereas the ROC calculated from the univariate ROC curve is often too optimistic. Considering the cost of testing, a multi-metabolites diagnostic model included two metabolites is recommended.

Some strengths and limitations of the current study should be acknowledged. First, this is the first untargeted metabolomics study of SS based on the plasma sample of Chinese Han people, allowing us to comprehensively evaluate the relationship between metabolites and SS. Second, we found that L-Glutamine and ChE 22:5n6 could perform as novel metabolic biomarkers. Moreover, EpiSS uses a rigorous protocol for measuring SS, provides enough objects for this study. Several potential limitations of this study should be mentioned. The small sample size of the sub sample of EpiSS metabolomics study may limit our ability to reveal metabolites with relatively small effect size, which is the major limitation of this study. The accuracy of the acute saline load test protocol is slightly lower than the dietary intervention protocol. On the other hand, different populations may show different metabolic characteristics due to the influence of individual growth and development level, although there are some similarities in the metabolite levels of the population. Finally, our results may differ from the previous untargeted metabolomics study of SS conducted among DASH-sodium study and GenSalt metabolomics study [19,20]. Most of the participants among DASH-sodium study were white or black, while our research participants are all Chinese Han descendants, following the common dietary pattern in northern China. The metabolomic analysis in GenSalt was conducted using urine biological samples, while this study used plasma for metabolomic study. Several studies reported the correlation of metabolite values through urine and serum [50,51], our study was different from GenSalt may be due to differences in biological samples.

## 5. Conclusions

In conclusion, our untargeted metabolomic in participants from the EpiSS showed that there were 39 DE-metabolites between SS and SR. L-Glutamine and Cholesterol ester 22:5n6 were identified as metabolic based biomarkers for SS. These data indicate that metabolic information may be an important tool to characterize SS. Moreover, our finding provides novel insights into the molecularly characterize of SSH.

## Figures and Tables

**Table 1:** Basic characteristics of the subjects in this study (*n* = 51).

Variable	Total (*n* = 51)	SS (*n* = 25)	SR (*n* = 26)	*p*
Age (years)	57.72 ± 5.42	57.71 ± 5.77	57.74 ± 5.06	0.980
Male (%)	15 (29.4)	8 (32.0)	7 (26.9)	0.691
BMI (kg/m^2^)	26.24 ± 3.48	26.56 ± 3.41	25.94 ± 3.53	0.536
Waist (cm)	88.76 ± 10.87	89.47 ± 10.15	88.08 ± 11.48	0.655
Hip (cm)	98.90 ± 7.96	100.63 ± 8.16	97.23 ± 7.39	0.132
FBG (mmol/L)	5.42 ± 0.57	5.48 ± 0.61	5.36 ± 0.52	0.460
TC (mmol/L)	5.47 ± 1.04	5.29 ± 1.08	5.64 ± 0.97	0.227
TG (mmol/L)	1.77 ± 0.89	1.94 ± 1.10	1.60 ± 0.58	0.174
LDL-C (mmol/L)	2.40 ± 0.96	2.06 ± 0.84	2.72 ± 0.98	0.012
HDL-C (mmol/L)	1.87 ± 1.11	2.00 ± 1.25	1.74 ± 0.93	0.417
Salt intake (g/day)	7.06 ± 4.48	6.74 ± 4.28	7.36 ± 4.64	0.626
Smoke (yes, %)	8 (15.7)	5 (20.0)	3 (11.5)	0.406
Drink (yes, %)	23 (45.1)	14 (56.0)	9 (34.6)	0.125
Hypertension (*n*, %)	26 (51.0)	14 (56.0)	12 (46.2)	0.482
Family history of Hypertension (*n*, %)	30 (58.8)	17 (68.0)	13 (50.0)	0.192
Anti-hypertension medication (*n*, %)	19 (37.2)	11 (44.0)	8 (30.8)	0.329
SBP (mmHg)	124.49 ± 21.61	114.60 ± 22.29	133.99 ± 16.29	0.001
DBP (mmHg)	79.65 ± 12.56	72.48 ± 10.93	86.54 ± 10.00	0.001

Abbreviations: SS, salt sensitive; SR, salt resistant; BMI, body mass index; TC, total cholesterol; TG, triglycerides; HDL-C, high-density-lipoprotein-cholesterol; LDL-C, low-density-lipoprotein-cholesterol; FBG, fasting blood glucose. *p* < 0.05 was considered statistically significant.

**Table 2:** Results of metabolites related regression models for SS.

Metabolites	Salt Sensitivity		
	OR	95% CI	*p*
TG 54:6	2.91	1.32–6.43	0.008
ChE 22:5n6	0.09	0.01–0.59	0.013
ChE 20:3	0.07	0.01–0.76	0.029
ChE 22:4	0.12	0.02–0.83	0.032
PC 32:1p	0.05	0.00–0.61	0.019
PC 16:1/14:0	0.43	0.19–0.97	0.043
PC 38:3e	0.05	0.01–0.48	0.009
Sphingosine 1-phosphate	4.17	1.46–1.90	0.008
AcCa 20:3	0.07	0.01–0.44	0.005
AcCa 20:2	0.20	0.04–0.89	0.035
AcCa 20:4	0.27	0.07–0.98	0.047
13S-hydroxyoctadecadienoic acid	2.74	1.23–6.09	0.014
Alpha-dimorphcolic acid	2.27	1.14–4.53	0.020
L-Glutamine	0.01	0.00–0.21	0.003
N (6)-Methyllysine	0.54	0.31–0.95	0.031
L-Lactic acid	0.12	0.02–0.63	0.012
L-Malic acid	0.07	0.01–0.58	0.013

Adjusted for age, gender, smoking, drinking, LDL−C, HDL−C and hypertension.

**Table 3:** Results of metabolites related regression models for Saline load period (_Δ_MAP_1_) and Diuresis shrinkage period (_Δ_MAP_2_).

Metabolites	_Δ_MAP_1_			_Δ_MAP_2_		
	Beta ^a^	SE	*p*	Beta ^b^	SE	*p*
TG 54:6	5.92	1.92	0.004	−4.65	1.38	0.002
ChE 22:5n6	−10.94	3.90	0.008	9.68	2.73	0.001
ChE 20:3	−12.01	5.95	0.050	9.53	4.33	0.033
ChE 22:4	−10.87	5.49	0.054	9.22	3.96	0.025
PC 32:1p	−14.44	6.42	0.030	10.85	4.69	0.026
PC 16:1/14:0	−1.87	1.40	0.189	1.96	1.00	0.057
PC 38:3e	−11.36	5.21	0.035	9.61	3.75	0.014
Sphingosine 1-phosphate	7.27	2.40	0.004	−3.26	1.88	0.089
AcCa 20:3	−13.94	3.91	0.001	6.81	3.10	0.034
AcCa 20:2	−10.72	4.46	0.021	3.88	3.44	0.265
AcCa 20:4	−8.19	3.43	0.022	3.57	2.62	0.182
13S-hydroxyoctadecadienoic acid	5.52	1.88	0.005	−2.83	1.45	0.057
Alpha-dimorphcolic acid	4.75	1.81	0.012	−2.56	1.38	0.070
L-Glutamine	−17.08	5.07	0.002	9.26	3.94	0.024
N (6)-Methyllysine	−3.85	1.60	0.021	2.30	1.20	0.063
L-Lactic acid	−9.21	3.94	0.024	4.62	2.99	0.130
L-Malic acid	−12.27	5.13	0.021	8.42	3.79	0.032

Adjusted for age, gender, smoking, drinking, LDL-C, HDL-C and hypertension. ^a^ Beta estimate corresponds to each metabolite during saline load period. ^b^ Beta estimate corresponds to each metabolite during diuresis shrinkage period.

**Table 4:** Comparison of R^2^ and C-statistics before and after addition of identified SS metabolites.

Metabolite	*R* ^2^	C-Statistics
Model1 ^a^	0.176	0.75
Model1 ^a^ + L−Glutamine + ChE 22:5n6	0.559	0.96
Difference	0.383	0.21
*p*	<0.001	<0.001

^a^ Model 1 was adjusted for age, gender, smoking, drinking, LDL-C, HDL-C and hypertension.

## Data Availability

Not applicable.

## Supplementary Materials

The following supporting information can be downloaded at: https://www.mdpi.com/article/10.3390/nu15030690/s1, Figure S1: Metabolic pathway analysis of differential metabolites between SS and SR; Table S1: Annotated plasma metabolites associated with SS relative to SR; Table S2: Metabolic pathway analysis of differential metabolites between SS and SR; Table S3: Results of metabolites related logistic regression models for SS; Table S4: ROC analysis of SS.
