# Supplementary material for: Association of Plasma Metabolites and Salt Sensitivity of Blood Pressure in Chinese Population: The EpiSS Study
Source: Nutrients. 2023 Jan 30;15(3):690. doi: 10.3390/nu15030690 (PMC9921558; doi:10.3390/nu15030690)
Supplement: Supplementary file 1 [file nutrients-15-00690-s001.zip › nutrients-2063164-supplementary-done.pdf]

nutrients

Supplementary Materials

Article

Association of Serum Metabolites and Salt Sensitivity of Blood Pressure in Chinese Population: The EpiSS Study

Fengxu Zhang †, Yunyi Xie †, Xiaojun Yang, Wenjuan Peng, Han Qi, Bingxiao Li, Fuyuan Wen, Pandi Li, Yuan Sun and Ling Zhang *

Table of Contents

Table S1. Annotated serum metabolites associated with SS relative to SR 2
Table S2. Metabolic pathway analysis of differential metabolites between SS and SR 4
Table S3. Results of metabolites related logistic regression models for SS. 6
Table S4. ROC analysis of SS. 8
Figure S1. Metabolic pathway analysis of differential metabolites between SS and SR 9

Table S1. Annotated serum metabolites associated with SS relative to SR. The results are based on the differential expression analysis on the comparisons of metabolites predicted to be differentially expressed based on cut-offs of adjusted p value < 0.05 and absolute log2 fold change > 0.3.

Metabolite	Super Pathway	Sub Pathway	*P*	Log2(FC)	VIP	Regulation
TG 54:6	Triacylglycerol	Triglyceride metabolism	0.006	0.80	2.08	Up
GlcCer d41:1	Glycosphingolipid	Sphingolipid metabolism	0.017	-0.31	1.07	Down
ChE 22:5n6	Cholesterol esters	Steroid biosynthesis	0.002	-0.65	1.92	Down
ChE 20:3	Cholesterol esters	Steroid biosynthesis	0.004	-0.48	1.62	Down
ChE 22:4	Cholesterol esters	Steroid biosynthesis	0.005	-0.48	1.54	Down
ChE 18:0	Cholesterol esters	Steroid biosynthesis	0.028	-0.52	1.45	Down
ChE 22:5n3	Cholesterol esters	Steroid biosynthesis	0.048	-0.33	1.13	Down
PC 32:1p	Glycerophosphocholine	Glycerophospholipid metabolism	0.004	-0.30	1.32	Down
PC 16:0/16:1	Glycerophosphocholine	Glycerophospholipid metabolism	0.007	-0.61	1.90	Down
PC 16:1/14:0	Glycerophosphocholine	Glycerophospholipid metabolism	0.007	-1.33	2.77	Down
PC 38:3e	Glycerophosphocholine	Glycerophospholipid metabolism	0.008	-0.35	1.29	Down
PC 17:1/14:0	Glycerophosphocholine	Glycerophospholipid metabolism	0.009	-0.93	2.26	Down
PC 16:0/18:1	Glycerophosphocholine	Glycerophospholipid metabolism	0.010	-0.31	1.34	Down
PC 30:0	Glycerophosphocholine	Glycerophospholipid metabolism	0.010	-0.61	1.90	Down
PC 30:1e	Glycerophosphocholine	Glycerophospholipid metabolism	0.010	-0.40	1.44	Down
PC 18:0p/14:0	Glycerophosphocholine	Glycerophospholipid metabolism	0.012	-0.31	1.16	Down
PC 14:0/18:2	Glycerophosphocholine	Glycerophospholipid metabolism	0.020	-0.40	1.34	Down
PC 15:0/14:0	Glycerophosphocholine	Glycerophospholipid metabolism	0.022	-0.86	2.11	Down
PC 17:1/16:0	Glycerophosphocholine	Glycerophospholipid metabolism	0.032	-0.38	1.36	Down
PC 20:4/22:6	Glycerophosphocholine	Glycerophospholipid metabolism	0.034	-0.37	1.38	Down
Sphingosine 1-phosphate	Phosphosphingolipids	Sphingolipid metabolism	0.009	0.59	1.60	Up
AcCa 20:3	Acylcarnitine	Fatty acid metabolism	0.002	-0.54	1.78	Down
AcCa 20:2	Acylcarnitine	Fatty acid metabolism	0.016	-0.37	1.30	Down
AcCa (5:0)	Acylcarnitine	Fatty acid metabolism	0.029	-0.32	1.00	Down

AcCa (20:4)	Acylcarnitine	Fatty acid metabolism	0.031	-0.43	1.38	Down
SM d36:0	Sphingomyelin	Sphingolipid metabolism	0.038	-0.39	1.24	Down
SM d22:0/16:0	Sphingomyelin	Sphingolipid metabolism	0.040	-0.34	1.22	Down
Cer d18:0/24:1	Ceramide	Sphingolipid metabolism	0.021	-0.34	1.14	Down
Cer d18:1/18:0	Ceramide	Sphingolipid metabolism	0.029	-0.38	1.14	Down
Cer d18:2/26:1	Ceramide	Sphingolipid metabolism	0.037	-0.32	1.22	Down
S-3-oxodecanoyl cysteamine	Fatty acid	Linoleic acid metabolism	0.013	-0.56	1.62	Down
13(S)-HODE	Fatty acid	Linoleic acid metabolism	0.020	1.11	2.37	Up
9(S)-HODE	Fatty acid	Linoleic acid metabolism	0.025	1.07	2.34	Up
L-Glutamine	Amino acid	Arginine biosynthesis	0.006	-0.34	1.41	Down
N (6)-Methyllysine	Amino acid	Lysine metabolism	0.009	-1.34	2.62	Down
L-Lactic acid	Organic acid	Glycolysis metabolism	0.009	-0.45	1.54	Down
L-Malic acid	Organic acid	TCA Cycle	0.011	-0.30	1.34	Down
N1-Methyl-2-pyridone-5-carboxamide	Vitamin and Cofactor	Nicotinate and nicotinamide metabolism	0.022	-0.45	1.10	Down
Phenylalanyl-Tryptophan	Peptide	organic amino metabolism	0.048	-0.32	1.04	Down

9(S)-HODE, oxylipins 9-hydroxyoctadecadienoic acid; 13(S)-HODE, oxylipins 13-hydroxyoctadecadienoic acid.

Table S2. Metabolic pathway analysis of differential metabolites between SS and SR.

Pathways	Total Pathway Metabolites	Metabolite Hits	*P*	Impact
Sphingolipid metabolism	21	3	0.003	0.31
Pyruvate metabolism	22	3	0.004	0.32
Glyoxylate and dicarboxylate metabolism	32	3	0.011	0
Arginine biosynthesis	14	2	0.017	0.06
Citrate cycle (TCA cycle)	20	2	0.034	0.09
Phenylalanine, tyrosine and tryptophan biosynthesis	4	1	0.058	0.50
Alanine, aspartate and glutamate metabolism	28	2	0.063	0.11
Linoleic acid metabolism	5	1	0.072	0
D-Glutamine and D-glutamate metabolism	6	1	0.086	0
Nitrogen metabolism	6	1	0.086	0
Glycerophospholipid metabolism	36	2	0.098	0.11
Arginine and proline metabolism	38	2	0.107	0.11
Pyrimidine metabolism	39	2	0.112	0.02
Ubiquinone and other terpenoid-quinone biosynthesis	9	1	0.126	0
Tyrosine metabolism	42	2	0.127	0.14
Phenylalanine metabolism	10	1	0.139	0
Aminoacyl-tRNA biosynthesis	48	2	0.158	0
alpha-Linolenic acid metabolism	13	1	0.177	0
Butanoate metabolism	15	1	0.202	0
Nicotinate and nicotinamide metabolism	15	1	0.202	0
Glycerolipid metabolism	16	1	0.214	0.01
Glycolysis / Gluconeogenesis	26	1	0.324	0.10
Glutathione metabolism	28	1	0.344	0
Glycine, serine and threonine metabolism	33	1	0.392	0
Cysteine and methionine metabolism	33	1	0.392	0

Arachidonic acid metabolism	36	1	0.420	0
Steroid biosynthesis	42	1	0.470	0
Purine metabolism	65	1	0.629	0
Steroid biosynthesis	42	1	0.527	0

Table S3. Results of metabolites related logistic regression models for SS.

**Metabolite**	**Model 1**			**Model 2**			**Model 3**			**Model 4**		
	**OR**	**95% CI**	***P***	**OR**	**95% CI**	***P***	**OR**	**95% CI**	***P***	**OR**	**95% CI**	***P***
TG 54:6	0.31	(0.05,1.9)	0.205	0.27	(0.04,1.73)	0.168	0.29	(0.05,1.83)	0.187	0.24	(0.04,1.6)	0.140
GlcCer d41:1	0.09	(0.01,0.59)	**0.013**	0.09	(0.01,0.63)	**0.015**	0.08	(0.01,0.55)	**0.010**	0.08	(0.01,0.58)	**0.013**
ChE 22:5n6	0.07	(0.01,0.76)	**0.029**	0.06	(0.01,0.7)	**0.024**	0.06	(0.01,0.75)	**0.029**	0.05	(0,0.66)	**0.024**
ChE 20:3	0.12	(0.02,0.83)	**0.032**	0.13	(0.02,0.94)	**0.043**	0.08	(0.01,0.69)	**0.021**	0.09	(0.01,0.79)	**0.030**
ChE 22:4	0.45	(0.15,1.36)	0.158	0.43	(0.14,1.32)	0.140	0.43	(0.14,1.32)	0.141	0.40	(0.12,1.26)	0.117
ChE 18:0	0.77	(0.19,3.18)	0.721	0.77	(0.19,3.2)	0.720	0.73	(0.17,3.04)	0.665	0.71	(0.17,3.02)	0.640
ChE 22:5n3	0.05	(0,0.61)	**0.019**	0.03	(0,0.48)	**0.014**	0.03	(0,0.51)	**0.014**	0.02	(0,0.4)	**0.010**
PC 32:1p	0.32	(0.1,1.02)	0.055	0.28	(0.08,0.94)	**0.040**	0.30	(0.09,0.99)	**0.049**	0.25	(0.07,0.88)	**0.031**
PC 16:0/16:1	0.43	(0.19,0.97)	**0.043**	0.40	(0.17,0.94)	**0.035**	0.41	(0.18,0.93)	**0.033**	0.37	(0.15,0.87)	**0.023**
PC 16:1/14:0	0.05	(0.01,0.48)	**0.009**	0.04	(0,0.42)	**0.007**	0.06	(0.01,0.49)	**0.009**	0.04	(0,0.43)	**0.007**
PC 38:3e	0.50	(0.23,1.07)	0.075	0.47	(0.21,1.04)	0.062	0.47	(0.21,1.04)	0.063	0.42	(0.18,0.99)	**0.047**
PC 17:1/14:0	0.23	(0.04,1.4)	0.112	0.22	(0.04,1.36)	0.102	0.23	(0.04,1.41)	0.112	0.22	(0.03,1.37)	0.104
PC 16:0/18:1	0.44	(0.16,1.21)	0.111	0.41	(0.14,1.18)	0.097	0.41	(0.15,1.15)	0.090	0.37	(0.12,1.1)	0.073
PC 30:0	0.26	(0.05,1.31)	0.102	0.20	(0.03,1.16)	0.073	0.22	(0.04,1.18)	0.077	0.14	(0.02,0.96)	**0.046**
PC 30:1e	0.19	(0.03,1.24)	0.082	0.13	(0.02,0.99)	**0.049**	0.17	(0.02,1.16)	0.070	0.11	(0.01,0.9)	**0.039**
PC 18:0p/14:0	0.52	(0.13,2.08)	0.357	0.50	(0.12,1.99)	0.322	0.54	(0.13,2.17)	0.384	0.51	(0.12,2.07)	0.343
PC 14:0/18:2	0.65	(0.36,1.19)	0.163	0.64	(0.35,1.18)	0.150	0.63	(0.34,1.16)	0.141	0.61	(0.32,1.14)	0.123
PC 15:0/14:0	0.49	(0.15,1.59)	0.237	0.47	(0.14,1.56)	0.220	0.47	(0.14,1.54)	0.211	0.44	(0.13,1.5)	0.189

PC 17:1/16:0	0.39	(0.11,1.32)	0.129	0.39	(0.11,1.39)	0.147	0.36	(0.1,1.27)	0.111	0.37	(0.1,1.34)	0.129
PC 20:4/22:6	4.17	(1.46,11.9)	**0.008**	4.11	(1.43,11.8)	**0.009**	4.10	(1.46,11.49)	**0.007**	4.03	(1.44,11.29)	**0.008**
Sphingosine 1-phosphate	0.07	(0.01,0.44)	**0.005**	0.06	(0.01,0.41)	**0.004**	0.07	(0.01,0.45)	**0.005**	0.06	(0.01,0.4)	**0.004**
AcCa 20:3	0.20	(0.04,0.89)	**0.035**	0.18	(0.04,0.84)	**0.030**	0.20	(0.04,0.91)	**0.038**	0.18	(0.04,0.87)	**0.033**
AcCa 20:2	0.33	(0.07,1.52)	0.154	0.24	(0.05,1.25)	0.090	0.35	(0.07,1.62)	0.179	0.25	(0.05,1.34)	0.106
AcCa (5:0)	0.27	(0.07,0.98)	**0.047**	0.25	(0.07,0.95)	**0.042**	0.25	(0.07,0.95)	**0.042**	0.23	(0.06,0.9)	**0.035**
AcCa (20:4)	0.53	(0.18,1.62)	0.266	0.36	(0.1,1.29)	0.116	0.54	(0.18,1.66)	0.284	0.32	(0.08,1.26)	0.104
SM d36:0	0.41	(0.1,1.72)	0.223	0.23	(0.04,1.25)	0.089	0.43	(0.1,1.85)	0.259	0.23	(0.04,1.27)	0.092
SM d22:0/16:0	0.23	(0.05,1.05)	0.057	0.17	(0.03,0.83)	**0.029**	0.20	(0.04,0.96)	**0.044**	0.13	(0.02,0.69)	**0.017**
Cer d18:0/24:1	0.50	(0.15,1.68)	0.259	0.36	(0.09,1.42)	0.147	0.49	(0.14,1.68)	0.257	0.33	(0.08,1.33)	0.119
Cer d18:1/18:0	0.32	(0.07,1.38)	0.127	0.26	(0.06,1.18)	0.082	0.31	(0.07,1.38)	0.123	0.25	(0.05,1.16)	0.077
Cer d18:2/26:1	0.28	(0.08,1.03)	0.055	0.29	(0.08,1.08)	0.064	0.25	(0.07,0.97)	**0.046**	0.26	(0.07,1.03)	0.054
S-3-oxodecanoyl cysteamine	2.74	(1.23,6.09)	**0.014**	3.21	(1.31,7.87)	**0.011**	2.70	(1.22,6.01)	**0.015**	3.27	(1.31,8.12)	**0.011**
13(S)-HODE	2.27	(1.14,4.53)	**0.020**	2.58	(1.21,5.49)	**0.014**	2.25	(1.13,4.47)	**0.021**	2.60	(1.22,5.53)	**0.013**
9(S)-HODE	0.01	(0,0.21)	**0.003**	0.01	(0,0.23)	**0.003**	0.01	(0,0.2)	**0.003**	0.01	(0,0.22)	**0.004**
L-Glutamine	0.54	(0.31,0.95)	**0.031**	0.56	(0.32,0.98)	**0.042**	0.52	(0.29,0.93)	**0.027**	0.54	(0.3,0.97)	**0.038**
N (6)-Methyllysine	0.12	(0.02,0.63)	**0.012**	0.13	(0.02,0.66)	**0.014**	0.11	(0.02,0.57)	**0.009**	0.11	(0.02,0.59)	**0.010**
L-Lactic acid	0.07	(0.01,0.58)	**0.013**	0.07	(0.01,0.63)	**0.017**	0.07	(0.01,0.54)	**0.011**	0.07	(0.01,0.6)	**0.015**
L-Malic acid	0.45	(0.17,1.23)	0.119	0.28	(0.09,0.9)	**0.033**	0.48	(0.17,1.32)	0.153	0.30	(0.09,0.97)	**0.045**
N1-Methyl-2-pyridone-5-carboxamide	0.30	(0.07,1.33)	0.113	0.30	(0.07,1.37)	0.120	0.31	(0.07,1.36)	0.120	0.30	(0.06,1.43)	0.132
Phenylalanyl-Tryptophan	0.89	(0.36,2.19)	0.806	0.89	(0.35,2.23)	0.796	0.87	(0.35,2.18)	0.770	0.84	(0.32,2.22)	0.729

Model1 adjusted for age, gender, smoking, drinking, LDL-C, HDL-C and hypertension. Model2 adjusted for age, gender, smoking, drinking, LDL-C, HDL-C, hypertension and BMI. Model3 adjusted for age, gender, smoking, drinking, LDL-C, HDL-C, hypertension and salt intake. Model4 adjusted for age, gender, smoking, drinking, LDL-C, HDL-C, hypertension, BMI and salt intake. Bold indicates the significance level is 0.05 for SS, saline load period and diuresis shrinkage period.

Table S4. ROC analysis of SS.

Metabolite	AUC	95% CI	*P*	Sensitivity	95% CI	Specificity	95% CI
**single-metabolite models**							
TG 54:6	0.83	(0.71,0.95)	<0.001	0.76	(0.59,0.93)	0.89	(0.76,1.00)
ChE 22:5n6	0.82	(0.7,0.93)	<0.001	0.6	(0.41,0.79)	0.92	(0.82,1.00)
ChE 20:3	0.82	(0.7,0.94)	<0.001	0.92	(0.81,1.00)	0.62	(0.43,0.8)
ChE 22:4	0.79	(0.67,0.92)	<0.001	0.8	(0.64,0.96)	0.69	(0.52,0.87)
PC 32:1p	0.81	(0.68,0.93)	<0.001	0.56	(0.37,0.76)	0.96	(0.89,1.00)
PC 16:1/14:0	0.79	(0.67,0.92)	<0.001	0.96	(0.88,1.00)	0.54	(0.35,0.73)
PC 38:3e	0.83	(0.72,0.95)	<0.001	0.72	(0.54,0.9)	0.85	(0.71,0.99)
Sphingosine 1-phosphate	0.82	(0.71,0.94)	<0.001	0.92	(0.81,1.00)	0.62	(0.43,0.8)
AcCa 20:3	0.84	(0.73,0.95)	<0.001	0.72	(0.54,0.9)	0.89	(0.76,1.00)
AcCa 20:2	0.81	(0.68,0.93)	<0.001	0.84	(0.7,0.98)	0.77	(0.61,0.93)
AcCa 20:4	0.81	(0.68,0.93)	<0.001	0.8	(0.64,0.96)	0.81	(0.66,0.96)
13S-hydroxyoctadecadienoic acid	0.81	(0.69,0.94)	<0.001	0.96	(0.88,1.00)	0.65	(0.47,0.84)
Alpha-dimorphcolic acid	0.81	(0.68,0.93)	<0.001	0.96	(0.88,1.00)	0.58	(0.39,0.77)
L-Glutamine	0.88	(0.78,0.97)	<0.001	0.88	(0.75,1.00)	0.77	(0.61,0.93)
N (6)-Methyl lysine	0.80	(0.67,0.92)	<0.001	0.92	(0.81,1.00)	0.58	(0.39,0.77)
L-Lactic acid	0.83	(0.71,0.94)	<0.001	0.76	(0.59,0.93)	0.77	(0.61,0.93)
L-Malic acid	0.81	(0.7,0.93)	<0.001	0.96	(0.88,1.00)	0.54	(0.35,0.73)
**multi-metabolites model**							
ChE 22:5n6	0.96	(0.91,1.00)	<0.001	0.96	(0.88,1.00)	0.85	(0.71,0.98)
L-Glutamine							

Adjusted for age, gender, smoking, drinking, LDL-C, HDL-C and hypertension.

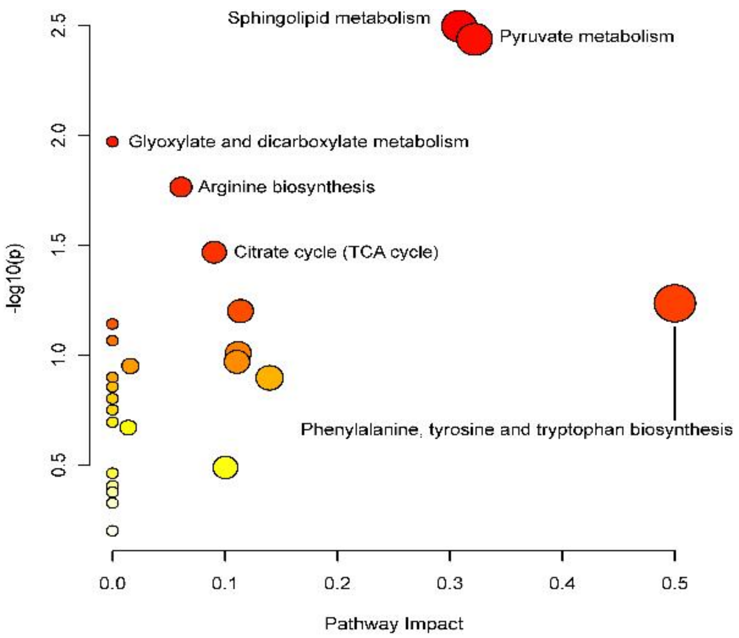

Figure S1. Metabolic pathway analysis of differential metabolites between SS and SR.
